# Physiological/pathological ramifications of transcription factors in the unfolded protein response

**DOI:** 10.1101/gad.297374.117

**Published:** 2017-07-15

**Authors:** Jaeseok Han, Randal J. Kaufman

**Affiliations:** 1 Soonchunhyang Institute of Medi-Bio Science (SIMS), Soonchunhyang University, Cheonan-si, Choongchungnam-do 31151, Republic of Korea;; 2 Degenerative Diseases Program, Sanford Burnham Prebys Medical Discovery Institute, La Jolla, California, 92307 USA

**Keywords:** ER stress, transcription factors, unfolded protein response

## Abstract

Numerous environmental, physiological, and pathological insults disrupt protein-folding homeostasis in the endoplasmic reticulum (ER), referred to as ER stress. Eukaryotic cells evolved a set of intracellular signaling pathways, collectively termed the unfolded protein response (UPR), to maintain a productive ER protein-folding environment through reprogramming gene transcription and mRNA translation. The UPR is largely dependent on transcription factors (TFs) that modulate expression of genes involved in many physiological and pathological conditions, including development, metabolism, inflammation, neurodegenerative diseases, and cancer. Here we summarize the current knowledge about these mechanisms, their impact on physiological/pathological processes, and potential therapeutic applications.

## Abstract (precis)

The unfolded protein response (UPR) is largely dependent on transcription factors that modulate expression of genes involved in many physiological and pathological conditions, including development, metabolism, inflammation, neurodegenerative diseases, and cancer. In this review, Han and Kaufman summarize the current knowledge about these mechanisms, their impact on physiological/pathological processes, and potential therapeutic applications.

The endoplasmic reticulum (ER) is the cellular organelle for protein folding and maturation, lipid and sterol biosynthesis, and calcium storage. ER homeostasis is disrupted by a number of insults that cause the accumulation of unfolded or misfolded proteins in the ER lumen, thereby activating the unfolded protein response (UPR) (Schroder and Kaufman 2005; Bernales et al. 2006). The UPR has outputs designed to couple the ER protein-folding capacity with demand so that the cell can survive and function. In order to increase protein-folding capacity, the homeostatic UPR expands the dimensions of the ER through increased biogenesis of protein and lipid components, including the protein translocation machinery, proteins that buffer folding reactions (chaperones), and trafficking machinery. Concurrently, the combined outputs of the homeostatic UPR increase transcription of ER-resident enzymes and structural components that increase protein-folding capacity and lead to the removal and degradation of misfolded proteins from the ER lumen in processes termed ER-associated degradation (ERAD) and macroautophagy (referred to here as autophagy). Of note are the penultimate effector transcription factors (TFs) in UPR signaling that activate or inhibit expression of target genes. Given the importance of TFs in the UPR, it is necessary to understand how these TFs function. Here, we describe the role of TFs involved in the UPR and how they contribute to human pathologies (Wang and Kaufman 2016).

## ER stress and the UPR

The ER is the site where proteins destined for the cell surface and the endomembrane system enter the secretory pathway (Kaufman 1999). Approximately one-third of all proteins are translocated across the ER membrane in an unfolded state, where they subsequently fold into their proper three-dimensional structures and are subject to glycosylation, hydroxylation, lipidation, and disulfide bond formation (Kaufman 1999, 2002; Ron 2002). The ER contains a high Ca^+2^ concentration and is occupied by chaperone proteins and enzymes that facilitate folding and post-translational modifications (Schroder and Kaufman 2005). Only properly folded proteins traffic to the Golgi compartment for further processing before transport to their final destination. Protein folding in the ER is disrupted by numerous insults, including pharmacological perturbations, genetic mutation of ER chaperones or their client proteins, elevated expression of proteins that transit the endomembrane system, viral infection, alterations in Ca^2+^ or redox status, differentiation of cells that secrete large amounts of proteins, and decreases as well as increases in available nutrients. The accumulation of unfolded or misfolded proteins in the ER lumen activates the UPR (Schroder and Kaufman 2005; Bernales et al. 2006). The UPR is signaled through three ER transmembrane proteins: inositol-requiring enzyme 1α (IRE1α), PKR (dsRNA-activated protein kinase)-related ER protein kinase (PERK), and activating TF 6α (ATF6α) (Scheuner and Kaufman 2008; Walter and Ron 2011). All three UPR sensors are maintained in an inactive state through interaction between their ER luminal domains and the protein chaperone immunoglobulin heavy chain-binding protein (BiP; also known as GRP78 and HSP5A). Upon ER stress and loss of ER homeostasis, accumulated unfolded/misfolded proteins in the ER lumen bind and sequester BiP, thereby promoting dissociation of BiP from IRE1α, PERK, and ATF6α (Bertolotti et al. 2000; Shen et al. 2002; Ali et al. 2011). ER stress sensors that are dissociated from BiP induce their downstream TFs through unique mechanisms described below.

## Activation of TFs in the UPR

**Figure 1.**
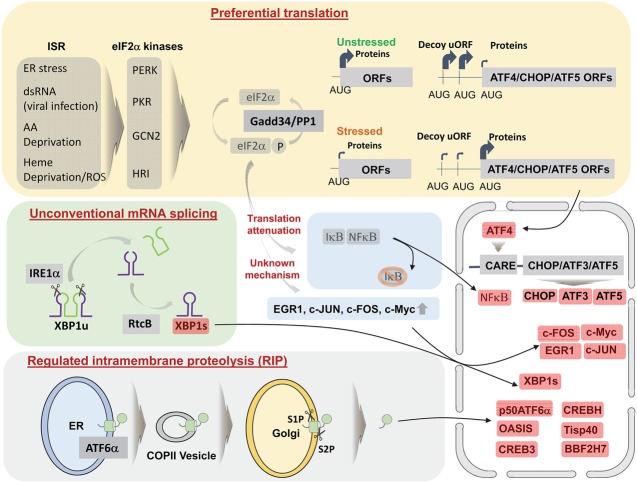
Activation of TFs in the UPR. Upon stresses, activated eIF2α kinases phosphorylate eIF2α that is dephosphorylated Gadd34/PP1. Although phosphorylated eIF2α attenuates general mRNA translation, some TFs, including ATF4, CHOP, and ATF5, are preferentially translated. ATF4 then translocates into the nucleus to activate the promoter region harboring CARE motifs. During translational attenuation, IκB, an inhibitor of NF-κB, is depleted (due to its short half-life) to activate NF-κB. In addition, eIF2α phosphorylation induces some TFs involved in the immediate early response, including EGR1, c-JUN, c-FOS, and c-Myc, through an unknown mechanism. XBP1 mRNA cleaved by IRE1α is ligated by RTCB to generate functional XBP1s mRNA. Unlike PERK and IRE1α, ATF6α released from BiP translocates to the Golgi apparatus through COPII vesicles, where the cytoplasmic region is cleaved by site-1 protease (S1P) and S2P. CREBH (cAMP response element-binding protein H), OASIS (old astrocyte specifically induced substance), Tisp40 (transcript induced in spermiogenesis), CREB3, and BBF2H7 (BBF2 human homolog on chromosome 7) are also activated through this mechanism.

In response to ER stress, the cell undergoes vast transcriptional reprograming by inducing or activating TFs. Following activation of proximal ER stress transducers, the activities of basic leucine zipper (bZIP)-containing TFs increase though preferential translation (e.g., ATF4), unconventional mRNA splicing (e.g., XBP1), or regulated intramembrane proteolysis (RIP; e.g., ATF6α) (Fig. 1). Other TFs in the UPR, including ATF3, CHOP (C/EBP [CCAT enhancer-binding protein] homologous protein), and ATF5, are induced through either preferential translation or conventional transactivation by ATF4, which binds to C/EBP ATF response elements (CAREs) in the promoter regions of target genes (Kilberg et al. 2009). The activities of additional TFs, including NFκB, increase due to a reduction in inhibitor levels as a consequence of translational attenuation mediated by eukaryotic initiation factor 2α (eIF2α) phosphorylation (Jiang et al. 2003; Deng et al. 2004). Some TFs, including c-JUN, c-FOS, EGR-1, and c-MYC, known as immediate early genes, are induced at very early time points after eIF2α phosphorylation, but their functions and induction mechanisms are unknown (Liang et al. 2006b).

### Preferential mRNA translation

During eukaryotic mRNA translation, the small ribosomal subunit (40S) is preloaded with Met-tRNAi by the GTP-bound form of eIF2 to form a 43S preinitiation complex (PIC). The 43S PIC binds the 5′ end of the mRNA and scans the 5′ untranslated region (UTR) until it encounters an AUG codon in a favorable Kozak consensus context (Kozak 1991) joining to a 60S ribosomal subunit coupled with eIF2-mediated GTP hydrolysis to GDP. Conversion of eIF2 to its GDP-bound state reduces its affinity for Met-tRNAi, causing it to dissociate from the PIC for recycling. To perform another round of initiation, eIF2B is required to promote GTP exchange for GDP on eIF2. Phosphorylation at Ser51 in eIF2α greatly increases the affinity of eIF2 for GDP, thereby preventing the eIF2B catalyzed exchange reaction and sequestering eIF2B with eIF2 in an inactive complex, resulting in global attenuation of mRNA translation. Paradoxically, translation of several mRNAs, including ATF4, is preferentially enhanced due to the presence of upstream ORFs (uORFs) (Harding et al. 2000; Kaufman 2004; Lu et al. 2004; Vattem and Wek 2004). ATF4 mRNA encodes a uORF (uORF1) for a three-amino-acid peptide, and the second uORF (uORF2) encodes a 59-amino-acid residue peptide that overlaps with the first 83 nucleotides (nt) of the ATF4-coding region (Vattem and Wek 2004). After synthesis of the uORF1-encoded polypeptide, ribosomes continue scanning along the ATF4 mRNA. When eIF2α-GTP is highly available in the absence of phosphorylated eIF2α, ribosomes reinitiate translation at uORF2, which overlaps out of frame with a portion of the ATF4-coding region, thereby reducing ATF4 translation. During ER stress conditions, the reduced amounts of available eIF2α-GTP permit an increase in time for scanning ribosomes to reinitiate, causing scanning ribosomes to bypass the initiation codon of the inhibitory uORF2. Thus, the scanning ribosomes associate with available eIF2α-GTP–Met-tRNAi to allow enhanced translation of ATF4. Although translation of some mRNAs, including *CHOP* (Palam et al. 2011), *GADD34* (Lee et al. 2009), and *ATF5* (Watatani et al. 2008), is up-regulated upon eIF2α phosphorylation, the mechanisms appear different from that of ATF4. For example, the 5′ UTR of *CHOP* has one uORF with a poor Kozak initiation context that might be bypassed by scanning ribosomes upon eIF2α phosphorylation. Consequently, scanning ribosomes would initiate at the *CHOP*-coding region that has a strong Kozak motif (Palam et al. 2011). Nevertheless, almost half of human transcripts contain one or more uORFs, suggesting that translational regulation at the initiation step has a pivotal role in the cellular response to ER stress (Barbosa et al. 2013; Hinnebusch et al. 2016). Indeed, a report describing the translational landscape in cancer indicated that translation from unconventional AUG codons may significantly impact cancer initiation (Sendoel et al. 2017). In addition, the potential for therapeutic intervention by targeting uORF translation was demonstrated recently (Liang et al. 2016).

### eIF2α kinases

There are four protein kinases that are dedicated to phosphorylating eIF2α at Ser51 (Fig. 1). The first identified was the heme-regulated inhibitory kinase (HRI) described in reticulocyte lysates. It functions to inhibit protein synthesis in reticulocytes upon heme deprivation in order to prevent misfolding of globin (Han et al. 2001). The second is the general control nonderepressible kinase (GCN2) that is activated by uncharged tRNAs to couple protein synthesis with amino acid availability (Sood et al. 2000). The third is PKR that is activated by dsRNA to prevent viral replication as part of the interferon response (Williams 1999). Finally, PERK evolved to respond to the accumulation of misfolded proteins in the ER to inhibit further production of misfolded proteins (Harding et al. 1999). The sum of these responses, all of which regulate translation through phosphorylation of eIF2α, was termed the integrated stress response (ISR). However, since different stresses all converge on eIF2α-P, increased mRNA translation cannot be assumed to be a consequence of ER stress.

#### Unconventional mRNA splicing

To obtain functional transcriptional activity, *XBP1* or yeast *HAC1* mRNA requires splicing initiated by IRE1. Upon activation by ER stress, IRE1 is autophosphorylated, which elicits its RNase activity to cleave *HAC1* or *XBP1* mRNA. While translation of unspliced *HAC1* mRNA is blocked by its intron in yeast, in metazoans, unspliced *XBP1* mRNA is efficiently translated to produce XBP1u, which binds the active TF XBP1s to enhance its degradation (Tirosh et al. 2006; Yoshida et al. 2006). In contrast to conventional splicing, which is catalyzed by the spliceosome and involves a consensus sequence at the exon and intron border junctions, such as GU–AG or AU–AC (Tarn and Steitz 1997), splicing of *HAC1* and *XBP1* is composed of a two-step unconventional splicing reaction. In yeast cells (*Saccharomyces cerevisiae*), activated Ire1p cleaves the unspliced *HAC1* mRNA at two RNA stem–loops to excise an intervening 252-base intron, and then the tRNA ligase Trl1p joins the two exons followed by removal of the junctional 2′ phosphate in the second step by 2′ phosphotransferase Tpt1p, generating the spliced form of *HAC1* mRNA (Sidrauski et al. 1996; Sidrauski and Walter 1997; Schwer et al. 2004). Similarly, in metazoans, IRE1α first removes a 23-nt (*Caenorhabditis elegans* and *Drosophila melanogaster*) or 26-nt (mammals) intron from the unspliced *XBP1* mRNA (Tirasophon et al. 1998; Shen et al. 2001; Yoshida et al. 2001; Calfon et al. 2002), and the proximally located tRNA ligase RTCB joins the two cleaved *XBP1* exons to generate a mature mRNA to produce the spliced form of XBP1 (Kosmaczewski et al. 2014; Lu et al. 2014). XBP1 mRNA appears to be the only substrate for IRE1α for splicing, as sophisticated searches for other substrates have failed (Bai et al. 2014).

#### RIP

Processing of ATF6α is different from the mechanisms by which ATF4 and XBP1s are induced. ATF6α is a type II transmembrane protein composed of a luminal domain that senses protein misfolding and a cytoplasmic DNA-binding portion containing a bZIP domain and transcriptional activation domain (Haze et al. 1999). Upon release from BiP, Golgi localization signals in its ER luminal region are exposed, and then ATF6α translocates to the Golgi apparatus, where it is cleaved by Golgi-resident proteases—first site-1 protease (S1P) and then S2P—to release the N-terminal bZIP TF domain (p50ATF6α) (Haze et al. 1999; Ye et al. 2000; Chen et al. 2002; Shen et al. 2002). These are the same processing enzymes that cleave the sterol-regulated element-binding proteins (SREBPs). In addition to ATF6α, several bZIP TFs located in the ER membrane are regulated by RIP. Those TFs include the cAMP response element-binding protein H (CREBH or CREB3L3) (Zhang et al. 2006), old astrocyte specifically induced substance (OASIS) (Kondo et al. 2005), BBF2 human homolog on chromosome 7 (BBF2H7) (Kondo et al. 2007), transcript induced in spermiogenesis α/β (Tisp40α/β) (Nagamori et al. 2005), and Luman/CREB3 (Liang et al. 2006a).

## Diverse roles of UPR TFs

Deletion and/or forced expression studies in different cell types demonstrate that each of these TFs provides unique and essential functions in response to ER stress (Table 1). Deletion of either *Ire1*α or *Xbp1* causes embryonic lethality because they are largely important for differentiation of cell types that secrete large amounts of protein, such as plasma cells that produce antibodies (Reimold et al. 2001; Iwakoshi et al. 2003; Zhang et al. 2005). In contrast, *Atf6α* deletion has no apparent phenotype in mice in the absence of ER stress; however, these mice cannot adapt to protein misfolding in the ER (Wu et al. 2007). Thus, this arm likely promotes an adaptive response to acute ER stress. Genes activated by XBP1s and ATF6α have largely complementary and overlapping functions, including ER protein folding, degradation, and trafficking (Yamamoto et al. 2007). Finally, the TFs downstream from phosphorylated eIF2α, ATF4, and CHOP activate unique and overlapping sets of genes that induce expression of ER chaperones, ER protein degradation, amino acid metabolism, the antioxidant response, and restoration of protein synthesis. The latter can lead to cell death if protein misfolding persists (Han et al. 2013a). In most case studies, the role of these TFs in promoting ER homeostasis has been delineated; however, it remains largely unknown how basal levels of these TFs act in the absence of UPR activation. Here, we summarize fundamental and diverse roles of these TFs in pathological processes.

**Table 1.**
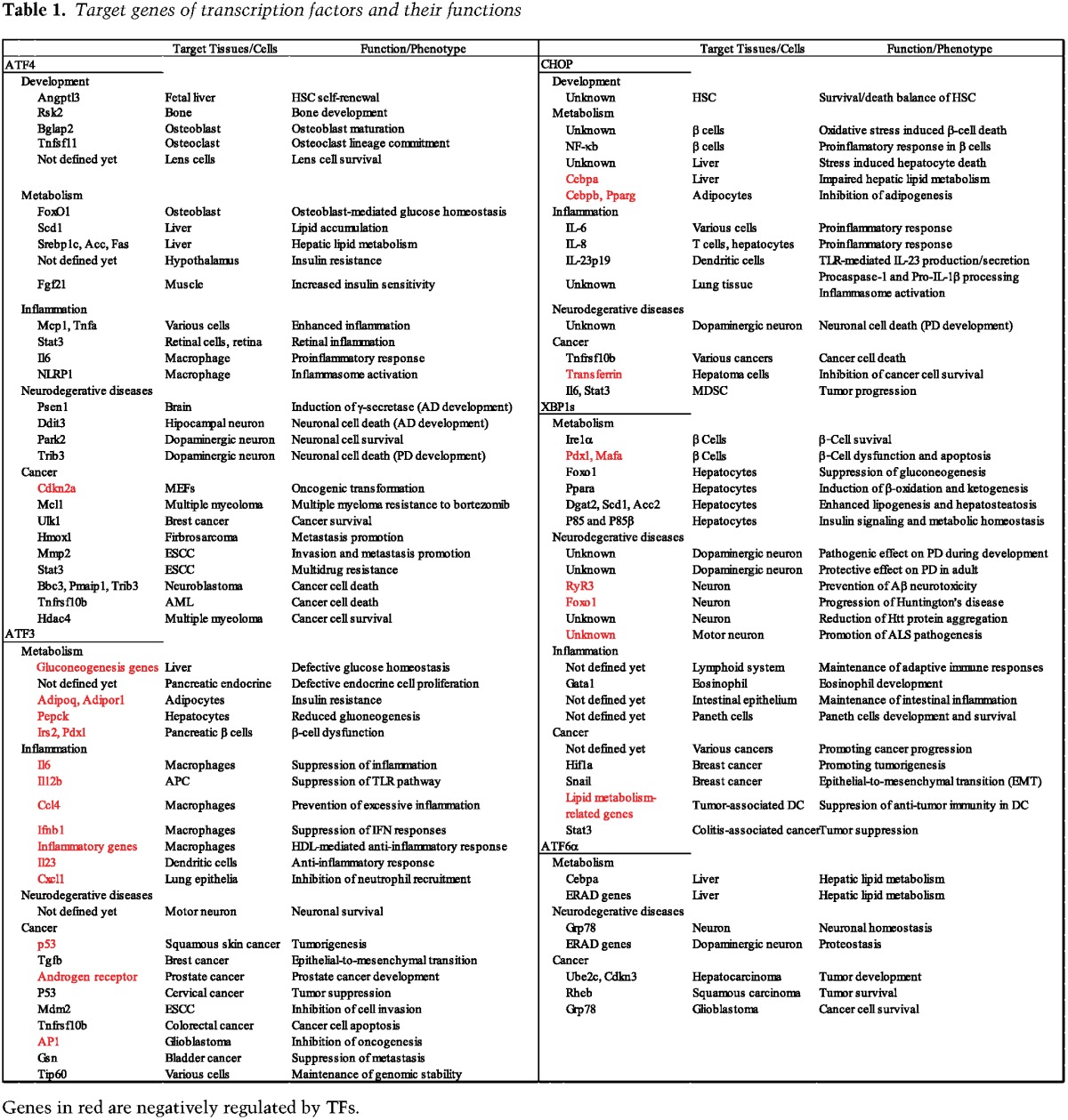
Target genes of transcription factors and their functions

### ATF4

#### Development

Studies suggest a pivotal role for ATF4 in maintaining stem cell integrity. *Atf4* deletion significantly impairs hematopoietic development and reduces hematopoietic stem cell (HSC) self-renewal due to decreased transcription of cytokine genes, including Angptl3 in fetal livers that serves as an important and unique site for rapid amplification of functional HSCs during development (Masuoka and Townes 2002; Zhao et al. 2015). Coffin-Lowry syndrome (CLS) is an X-linked mental retardation condition associated with skeletal abnormalities caused by a mutation in the protein kinase *RSK2* gene. ATF4 is a critical substrate of RSK2, and deletion of *Atf4* delays bone formation during embryonic development and decreases bone mass throughout postnatal life (Yang et al. 2004). ATF4 forms heterodimers with its critical partner, C/EBPβ, which is a bZIP TF, acts on the osteocalcin (*Bglap2*) promoter, and is essential for osteoblast maturation (Tominaga et al. 2008). ATF4 is positively regulated by a stress-activated protein kinase, JNK (Matsuguchi et al. 2009); fibroblast growth factor 2 (FGF2) (Fei et al. 2010); and the ubiquitous TF Forkhead box O1 (FoxO1) during osteoblast differentiation (Rached et al. 2010). Another study also suggests that microRNA miR-214 reduces the amount of ATF4 protein but not mRNA levels to inhibit osteoblast function (Wang et al. 2013a). ATF4 also regulates osteoclast differentiation and ultimately bone resorption through expression in osteoblasts. ATF4 binds to the promoter and activates expression of the receptor activator of NF-κB ligand (RANKL; *Tnfsf11*). RANKL is a factor secreted by osteoblasts that binds to its receptor (RANK) on osteoclasts to trigger intricate and distinct signaling cascades that control osteoclast lineage commitment and activation (Cao et al. 2010). Thereby, ATF4 promotes bone formation.

*Atf4*^−/−^ mice display microphthalmia due to a complete absence of the lens through massive and synchronous apoptosis of the anterior epithelial lens (Hettmann et al. 2000). ATF4 is expressed at high levels in the anterior epithelial lens cells at embryonic day 14.5. The defective lens formation in the absence of ATF4 is not due to qualitative defects in the expression of lens-specific genes, including Pax-6, αA-crystallin, c-Maf, or PDGF-Rα, but rather the death of *Atf4*^−/−^ epithelial lens cells is mediated by a p53-dependent apoptotic pathway, suggesting an essential role of ATF4 in retinal cell survival. However, overexpression of ATF4 in *Xenopus laevis* embryos interfered with neurogenesis and eye formation, suggesting that tightly controlled ATF4 expression may be crucial for normal eye patterning (Liu et al. 2011).

#### Metabolism

*Atf4*^−/−^ mice exhibit a lean phenotype and resistance to diet-induced obesity, with lower levels of circulating carbohydrates (Seo et al. 2009). Although *Atf4*^−/−^ mice did not exhibit an obvious defect in pancreatic β cells (Back et al. 2009), ATF4 seems to regulate glucose metabolism in mice by regulating osteoblast function (Kode et al. 2012). *Atf4* deletion in mice improved glucose and insulin sensitivity, which was abolished by overexpression of ATF4 in osteoblasts through cooperation with FoxO1 (Kode et al. 2012). Furthermore, *Atf4* deletion specifically in murine osteoblasts causes the same metabolic phenotypes as *Atf4*^−/−^ mice, suggesting a requirement of ATF4 in osteoblast-mediated glucose homeostasis (Yoshizawa et al. 2009).

ATF4 appears to promote liver steatosis. Mice fed a high-carbohydrate diet (HCD) accumulate hepatic triglycerides (TGs) and display impaired glucose tolerance, which is diminished in the absence of ATF4 (Li et al. 2011). In the livers of *Atf4*^−/−^ mice fed a HCD, stearoyl-CoA desaturase 1 (SCD1) expression is markedly lower than wild-type livers, and overexpression of ATF4 restores levels of SCD1 and increases hepatic lipid accumulation (Li et al. 2011). Lipid accumulation caused by a high-fructose diet (HFrD) is also attenuated in *Atf4*^−/−^ mice due to decreased levels of three key genes in the lipogenic pathway, including sterol regulatory element-binding protein 1c (SREBP1c), acetyl-CoA carboxylase (ACC), and fatty acid synthase (FAS), suggesting a role for ATF4 in promoting hepatic lipid accumulation in response to nutritional stimuli (Xiao et al. 2013).

An association between ATF4 and insulin sensitivity is also evident. Overexpression of ATF4 in the hypothalamus induces hepatic insulin resistance in mice, and inhibition of ATF4 by expressing dominant-negative ATF4 has the opposite effect. Furthermore, inhibition of ATF4 in the hypothalamus reverses insulin resistance caused by ER stress in the brain, suggesting that ER stress causes hepatic insulin resistance through ATF4 (Zhang et al. 2013). On the other hand, ATF4 increases insulin sensitivity in mice. Elimination of autophagy selectively in muscle reduces diet-induced obesity and insulin resistance by promoting ATF4-mediated induction of FGF21 expression (Kim et al. 2013). It is unclear why ATF4 expression in the hypothalamus causes insulin resistance but in the muscle increases insulin sensitivity.

#### Inflammation

ER stress and subsequent UPR activation are implicated in inflammatory responses that contribute substantially to disease progression (Zhang and Kaufman 2008; Hotamisligil 2010). In ER stress-mediated inflammation, ATF4 increases expression of inflammatory cytokines, including interleukin-6 (IL-6) and monocyte chemoattractant protein-1 (MCP-1), by inducing transcription of the NACHT, LRR, and PYD domain-containing protein 1 (NLRP1), a core component of the inflammasome. In response to ER stress, ATF4 binds to the promoter and induces expression of NLRP1, providing evidence that ATF4 induces an inflammatory response (D'Osualdo et al. 2015). ER stress caused by high glucose in endothelial cells induces inflammatory factors, including tumor necrosis factor-α (TNF-α) and MCP-1, which is reduced by inhibiting ATF4 activity (Caselli et al. 2012; Chen et al. 2012; Wang et al. 2014; Huang et al. 2015). Forced expression of ATF4 induces endothelial inflammation through activation of STAT3-mediated cytokine production. Down-regulation of ATF4 significantly attenuates retinal inflammation in type 1 diabetic models (Chen et al. 2012). ATF4 is also implicated in the saturated fatty acid (SFA)-induced IL-6 expression in macrophages (Iwasaki et al. 2014). Attenuation of ATF4 in macrophages markedly inhibits SFA-induced IL-6 expression, whereas forced expression of ATF4 enhances IL-6 expression through direct activation of the IL-6 promoter and/or activation of NF-κB (Iwasaki et al. 2014).

#### Neurodegenerative disease

ATF4 expression is increased in the hippocampus of Alzheimer's disease (AD) murine models and the axons from AD cadavers, suggesting a potential role of ATF4 in the spreading of AD pathology (Baleriola et al. 2014; Ohno 2014). ATF4 increases the expression of human presenilin-1 (PS1), an important subunit of the γ-secretase responsible for Aβ production during AD pathogenesis (Mitsuda et al. 2007). Local application of Aβ1–42 causes axonal synthesis of ATF4 and subsequent induction of CHOP, leading to neuronal cell death. This phenomenon was abolished by knockdown of ATF4 expression (Baleriola et al. 2014).

Treatment with 6-hydroxydopamine (6-OHDA), a drug that induces a Parkinson's-like disease (PD), increases expression of several UPR genes, including ATF4, suggesting a potential role for ER stress in PD (Holtz et al. 2005). One of contributing factors for PD development is loss of the E3 ubiquitin ligase Parkin in dopaminergic neurons. The expression of Parkin is induced by ER stress through direct binding of ATF4 to the promoter region of the *Parkin* gene (Bouman et al. 2011). Given the protective role of Parkin, these results suggest that ATF4 promotes dopaminergic cell survival during PD pathogenesis. Consistently, ATF4 levels are increased in neurons in the substantia nigra in a subset of PD patients compared with controls (Sun et al. 2013). In addition, ATF4 overexpression in cellular models of PD reduces cell death, whereas silencing of ATF4 enhances cell death caused by 6-OHDA. In contrast, tribbles pseudokinase 3 (Trib3), a proapoptotic factor in the UPR, is transcriptionally induced by ATF4 in a cellular PD model upon 6-OHDA treatment, suggesting a proapoptotic role for ATF4 in PD pathogenesis (Aime et al. 2015). Analysis of PD in mice with *Atf4* deletion in dopaminergic neurons should provide greater insight into the role of ATF4 in PD.

#### Cancer

Increased ATF4 expression was observed in murine and human tumor tissues (Ameri et al. 2004; Bi et al. 2005; Ye et al. 2010). Increased ATF4 expression facilitates tumorigenesis by modulating transcription of genes involved in tumor cell proliferation. ATF4 suppresses the expression of the cellular senescence-associated gene *Cdkn2a* (cyclin-dependent kinase inhibitor 2a) to drive oncogenic transformation (Horiguchi et al. 2012) and enhances expression of the anti-apoptotic gene myeloid cell leukemia-1 (*Mcl-1*) (Hu et al. 2012), the autophagy-initiating kinase *Ulk1* (Pike et al. 2013), and heme oxygenase 1 (*Hmox-1*) (Dey et al. 2015) to promote cancer cell survival. In addition, ATF4 causes cell invasion and metastasis by inducing matrix metalloproteinase 2 (*Mmp2*) (Zhu et al. 2014b). ATF4 also promotes multidrug resistance (MDR) expression, a major challenge to cancer treatment, through transactivation of signal transducer and activator of transcription 3 (*Stat3*) (Zhu et al. 2014a).

In contrast, other studies implicate ATF4 in an apoptotic response in tumors. Glutamine depletion selectively induces apoptosis in oncogenic MYC-overexpressing cells through ATF4-dependent induction of proapoptotic proteins PUMA and NOXA (Qing et al. 2012). The anti-cancer drug ONC201 induces tumor cell death through ATF4-mediated transactivation of the proapoptotic protein TRAIL and its receptor, death receptor 5 (DR5) (Ishizawa et al. 2016). ATF4-driven expression of CHOP is enhanced by a histone deacetylase (HDAC) inhibitor, thereby enhancing apoptosis upon proteasome inhibitor treatment (Kikuchi et al. 2015).

As discussed above, basal expression of ATF4 is indispensable for bone and eye development as well as metabolic homeostasis. The role of ATF4 upon ER stress is different in each tissue, likely due to the diversity of binding partners that form heterodimers under different conditions. In addition, the diversity of ATF4 downstream target genes might be another explanation for the different downstream responses. Therefore, identification of binding partners or target genes of ATF4 under different conditions will provide valuable insight toward understanding the complexities of the role of ATF4.

### ATF6α

#### Metabolism

ATF6α is essential for transcriptional induction of ER molecular chaperones as well as components of ERAD. Although *Atf6*α^−/−^ mice display no apparent developmental phenotype under normal growth conditions, *Atf6*α deletion severely impairs liver function and prolongs steatosis compared with wild-type mice upon ER stress (Wu et al. 2007; Rutkowski et al. 2008; Yamamoto et al. 2010). This might result from prolonged CHOP expression in response to chronic UPR activation and consequent suppression of C/EBPα (Rutkowski et al. 2008) as well as reduced expression of chaperones and ERAD functions (Wu et al. 2007; Yamamoto et al. 2010). On the other hand, forced expression of the functionally active nuclear fragment of ATF6 in zebrafish causes fatty liver (Howarth et al. 2014), suggesting that fine-tuning of ATF6α may be important to prevent liver steatosis.

The role of ATF6α in the pathogenesis of human disease is also evident in diabetes, particularly in insulin-producing pancreatic β cells. Single-nucleotide polymorphisms exist in a functionally important region of the *ATF6*α gene that is associated with type 2 diabetes in a population of Pima Indians (Thameem et al. 2006), Dutch Caucasians (Meex et al. 2007), and Chinese (Gonzalez-Rodriguez et al. 2014). Moreover, high-fat diet (HFD)-fed *Atf6*α^−/−^ mice displayed glucose intolerance, blunted insulin secretion, and reduced pancreatic insulin content due to β-cell failure (Usui et al. 2012). In type 1 diabetes, there is a progressive loss of ATF6α expression before the onset of diabetes in nonobese diabetic (NOD) mice as well as in pancreata from type 1 diabetic patients, suggesting that ATF6α protects β cells (Engin et al. 2013). Curiously, the diabetic phenotype in murine models was recovered by treatment with the chemical chaperone taurourosodeoxycholic acid (TUDCA). Chemical chaperones are proposed to buffer protein folding in the ER that reduces ER stress. However, the beneficial effect of TUDCA treatment in NOD mice was abolished in the absence of ATF6α specifically in β cells, suggesting that TUDCA protects β cells from ER stress-mediated cell death in an ATF6α-dependent manner. This intriguing result needs further investigation because if TUDCA prevents accumulation of misfolded proteins, it would not be expected to activate ATF6α. It was demonstrated recently that hypomorphic mutations in *ATF6*α in humans cause a rare syndrome, achromatopsia, that is associated with age-onset color blindness and loss of cone photoreceptors in the retina (Ansar et al. 2015; Kohl et al. 2015; Chiang et al. 2017). Intriguingly, ATF6α deletion did not affect the function of rod photoreceptors, indicating a very selective requirement for ATF6α in cone photoreceptors.

#### Neurodegenerative disease

ATF6α also plays an important role in neurodegeneration. For example, 6-OHDA-induced PD enhances activation of the PERK/eIF2α pathway as well as ATF6α, and *Atf6*α^−/−^ mice exhibit accelerated neuronal degeneration and ubiquitin accumulation due to reduced expression of BiP/GRP78, an ATF6α-dependent molecular chaperone in the ER (Hashida et al. 2012). Furthermore, impaired ATF6α signaling decreases ERAD function and increases proapoptotic signaling in PD animal models (Credle et al. 2015), suggesting that proteostasis maintained by ATF6α is critical to prevent PD.

#### Cancer

Elevated expression of ATF6α is observed in human hepatocellular carcinoma (Shuda et al. 2003). ATF6α transactivates target genes that include ubiquitin-conjugating enzyme E2C (*UBE2C*) and *CDKN3*, which promote tumorigenesis (Arai et al. 2006). Polymorphisms in *ATF6a* are associated with increased expression and hepatocellular carcinoma (Wu et al. 2014). On the other hand, ATF6α prolongs survival of dormant tumor cells, but not proliferative squamous carcinoma cells, through transactivation of the Ras homolog enriched in brain (*Rheb*; a critical activator of the mammalian target of rapamycin [mTOR]) and thus activation of mTOR signaling (Schewe and Aguirre-Ghiso 2008). Similarly, ATF6α protects glioblastoma cells from UV-induced cell death by transactivating BiP, suggesting proto-oncogenic effects of ATF6α. Finally, as BiP expression frequently correlates with tumor status, chemoresistance, and prognosis (Lee and Hendershot 2006; Wang and Kaufman 2014) and as ATF6α is the primary driver of BiP expression, targeting BiP expression via the ATFT6α pathway should be considered a therapeutic approach for cancer (Gutierrez and Simmen 2014; Obacz et al. 2017).

In contrast to other UPR TFs, ATF6α is not necessary to maintain the physiological state, since *Atf6a*^−/−^ mice do not exhibit overt phenotypes. The primary function of ATF6α is likely to protect cells from acute ER stress; however, its target genes are yet to be clearly identified due to the absence of suitable antibodies for ChIP-seq (chromatin immunoprecipitation [ChIP] combined with high-throughput sequencing) analyses. Future studies, including ChIP-seq and RNA sequencing (RNA-seq), will identify sets of genes regulated by ATF6α, which will provide insight into the function of ATF6α.

### ATF3

#### Metabolism

Overexpression of ATF3 inhibits expression of gluconeogenesis genes in the liver and also causes aberrations in the endocrine pancreas with reduced hormone-producing cells in the islets, resulting in defective glucose homeostasis (Allen-Jennings et al. 2002). ATF3 decreases expression of adiponectin (Kim et al. 2006) and the adiponectin receptor (Park et al. 2010a) in adipocytes, phosphoenolpyruvate carboxykinase (PEPCK) in hepatocytes (Allen-Jennings et al. 2002), and the insulin receptor substrate 2 (IRS2) (Li et al. 2008) and pancreatic and duodenal homeobox factor 1(PDX1), a key differentiation factor for pancreatic development (Jang et al. 2011), in pancreatic β-cells. Mice lacking ATF3 exhibit defects in β-cell function with reduced insulin secretion upon a HFD (Zmuda et al. 2010). Therefore, proper regulation of ATF3 expression appears critical to maintain differentiated cell function.

#### Inflammation

ATF3 is induced during Toll-like receptor (TLR)-dependent immune responses and represses expression of numerous proinflammatory cytokines, including IL-6 and IL-1β, by altering chromatin structure to restrict access to TFs such as NF-κB in macrophages (Gilchrist et al. 2006; Whitmore et al. 2007). ATF3 also modulates the expression of macrophage inflammatory protein 1 (MIP-1, also known as CCL4) in macrophages, thereby preventing excessive inflammation (Khuu et al. 2007). ATF3 decreases IFN responses by controlling basal and inducible levels of IFN-β and expression of IFN target genes in macrophages (Labzin et al. 2015). Thus, *Atf3*^−/−^ mice are more susceptible to endotoxic shock due to excessive cytokine production (Hoetzenecker et al. 2012). ATF3 also mediates high-density lipoprotein (HDL)-induced anti-inflammatory reprogramming of macrophages by transcriptional repression of inflammatory genes (De Nardo et al. 2014). In neutrophils, ATF3 restricts neutrophil recruitment by reducing neutrophil chemokine production that promotes neutrophil chemotaxis (Boespflug et al. 2014). ATF3 also suppresses the IL-23 pathway in dendritic cells to exert an IL-4-mediated anti-inflammatory effect (Whitmore et al. 2007; Guenova et al. 2015).

#### Neurodegenerative disease

Amyotrophic lateral sclerosis (ALS) is an adult-onset degenerative disorder of motor neurons. Intact adult motor neurons do not normally express ATF3. However, ATF3 expression is observed in spinal motor neurons in an ALS murine model that harbors a transgene-expressing human cytosolic superoxide dismutase 1 with an ALS-associated mutation (hSOD1G93A) (Malaspina et al. 2010). Interestingly, forced expression ATF3 promotes neuronal survival and delays the ALS phenotype in hSODG93A transgenic mice (Seijffers et al. 2014), suggesting that ATF3 is protective in ALS.

#### Cancer

Accumulating evidence suggests that ATF3 plays a pivotal role in cancer development by regulating the balance between survival and cell death. As a proto-oncogene, ATF3 expression is elevated in human breast cancer (Yin et al. 2008), malignant human prostate cancer (Pelzer et al. 2006), malignant Hodgkin's lymphoma (Janz et al. 2006), and squamous cell carcinoma (Wu et al. 2010). ATF3 reduces expression of tumor suppressor p53 and its downstream target genes in squamous cell carcinoma (Wu et al. 2010) and transactivates expression of TGFβ genes in breast cancer (Yin et al. 2010). In addition, ATF3 represses androgen-dependent genes by inhibiting androgen activity, resulting in prostate cancer development (Wang et al. 2012a).

In contrast to the above results, ATF3 expression is decreased in human colorectal cancer (Bottone et al. 2003), cervical cancer (Wang et al. 2010), and glioma (Gargiulo et al. 2013) compared with normal tissues, suggesting that ATF3 may act as a tumor suppressor. ATF3 activates p53 by preventing its ubiquitination and degradation in cervical cancer (Wang et al. 2010). ATF3 also increases expression of MDM2 to facilitate MMP-2 degradation and subsequent inhibition of cell invasion in esophageal squamous cell carcinoma (Xie et al. 2014). In colorectal cancer, ATF3 activates DR5 to enhance sensitivity to apoptotic cell death (Taketani et al. 2012; Edagawa et al. 2014). In addition, bone morphogenetic protein (BMP) signaling activates ATF3 to bind open chromatin structures at AP1-preloaded sites and inhibit the oncogenic network (Gargiulo et al. 2013). ATF3 also suppresses bladder cancer metastasis through promoting gelsolin-mediated actin remodeling (Yuan et al. 2013) and maintains genomic stability by activating ataxia telangiectasia mutated (ATM) signaling (Cui et al. 2015).

It is not clear how ATF3 acts in a dichotomous nature in cancer development. It is possible that stress-inducible ATF3 is involved in p53-dependent target gene expression and apoptosis, whereas tumor-related ATF3 suppresses proapoptotic genes in the p53 pathway (Taketani et al. 2012). These results suggest that ATF3 has cell context-dependent effects on p53 target genes in the stress response and cancer development. However, the exact mechanism of ATF3 in transformation and the role of p53 remain to be elucidated. As many tumors are p53-negative, this is an important question.

Although the function of ATF3 extends to other physiological responses, ATF3 appears to play a role mainly in inflammation and cancer. It is noteworthy that ATF3 expression is highly induced by not only ER stress but oxidative stress and DNA damage. It is not known whether this induction is mediated by eIF2α phosphorylation or P53. Thus, the role of ATF3 should be interpreted based on the combined effects of these stimuli.

### CHOP/DDIT3/GADD153

#### Development

Since *Chop*^−/−^ mice are born without developmental defects, it seems that CHOP is not necessary for embryonic development. However, HSCs from *Chop*^−/−^ mice exhibit increased viability, suggesting that CHOP may be required for the survival/death balance of mouse HSCs under physiological conditions (van Galen et al. 2014). The role of CHOP in development or differentiation of stem cells remains to be elucidated.

#### Metabolism

*Chop* deletion in β cells is protective in several diabetic murine models. In heterozygous *Akita* mice, which exhibit a diabetic phenotype due to a misfolding mutation (Cys96Tyr) in the insulin 2 gene, disruption of the *Chop* gene delays the onset of diabetes (Oyadomari et al. 2002). In both genetic (leptin receptor deficient *db/db* mice) and HFD-induced type 2 diabetic murine models, *Chop* deletion improves β-cell ultrastructure, function, and survival, suggesting that CHOP is a fundamental factor that links ER stress to apoptosis in β cells under conditions of increased insulin demand in type 2 diabetes (Song et al. 2008). *Chop* deletion also protects β cells from cytokine-induced proinflammatory responses by reducing cytokine-induced NF-κB activity (Allagnat et al. 2012). The exact mechanism by which CHOP mediates β-cell death is not clear, but evidence suggests that oxidative stress caused by ER protein misfolding significantly contributes to β-cell death (Song et al. 2008; Li et al. 2010; Han et al. 2013a, 2015).

CHOP is also involved in liver dysfunction upon ER stress. *Chop* deletion protects mice from various hepatocyte-specific challenges, including bile duct ligation (Tamaki et al. 2008), acetaminophen (Uzi et al. 2013), alcohol feeding (Ji et al. 2005), and diet-induced steatohepatitis (Rinella et al. 2011; Toriguchi et al. 2014). In contrast to the beneficial effect of *Chop* deficiency, *Chop*^−/−^ mice fed a methionine–choline-deficient (MCD) diet display increased liver damage (Soon et al. 2010), possibly explained by a net accumulation of activated macrophages due to decreased death in the absence of CHOP (Malhi et al. 2013). As all of these studies were performed with ubiquitous *Chop* deletion, mechanistic insight is limited.

In addition to cell death, CHOP also is involved in hepatic lipid metabolism. ER stress induces CHOP that suppresses C/EBPα activity and other lipid master regulatory genes (Rutkowski et al. 2008). Consistently, *Chop*^−/−^ mice exhibit less hepatic lipid accumulation than wild-type mice upon treatment with human immunodeficiency virus (HIV) protease inhibitors (Wang et al. 2013b).

CHOP is also involved in adipocyte differentiation. As a dominant-negative inhibitor of C/EBPα and C/EBPβ (Ron and Habener 1992), CHOP expression inhibits adipocyte differentiation under stress conditions (Batchvarova et al. 1995; Han et al. 2013b). It seems that CHOP sequesters and inhibits C/EBPβ activity to attenuate adipogenesis (Tang and Lane 2000). Transient CHOP expression in the early phase of differentiation of 3T3L1 cells (Tang and Lane 2000) completely inhibits adipogenesis (Han et al. 2013b), suggesting that strict regulation of CHOP is essential for adipocyte differentiation.

#### Inflammation

CHOP is involved in inflammatory processes through the regulation of cytokine expression. CHOP promotes *Il6* gene expression at the transcriptional level indirectly through dimerization with an inhibitory isoform of C/EBPβ (LIP) to prevent binding to the *Il6* promoter (Hattori et al. 2003). On the other hand, following prostaglandin stimulation of T cells, CHOP directly binds to and induces the *CXCL8* (also know as *IL8*) promoter (Cucinotta et al. 2008). Consistently, SFA-damaged hepatocytes secrete IL-8, which causes liver inflammation, contributing to the pathogenesis of nonalcoholic steatohepatitis (NASH) (Willy et al. 2015). CHOP binds to and induces expression of IL-23p19, a key mediator of inflammation in dendritic cells (Goodall et al. 2010). Infection of myeloid cells with bacteria induces CHOP transcription with subsequent induction of IL-23, which is greatly attenuated by knockdown of CHOP. In addition, activation of procaspase-1 and pro-IL-1β is attenuated by *Chop* deletion in LPS-treated lungs of mice due to impaired induction of caspase-11, suggesting that CHOP mediates ER stress-mediated inflammasome activation (Endo et al. 2006). Thus, CHOP appears to regulate the immune response at multiple levels in different cell types.

#### Neurodegenerative disease

CHOP expression is induced in human neuroblastoma SH-SY5Y cells in vitro as well as in substantia nigra dopaminergic neurons treated with 6-OHDA in vivo, suggesting that CHOP contributes to PD development (Silva et al. 2005; Yamamuro et al. 2006). Angiotensin II receptor blockade decreases dopaminergic cell death caused by 6-OHDA by down-regulating CHOP expression (Wu et al. 2013). In addition to PD, CHOP expression is also markedly induced in brains from mice with AD (Lee et al. 2010a). Silencing of CHOP in the rabbit hippocampus protects animals from AD induced by 27-hydroxycholesterol, an oxidized metabolite of cholesterol (Prasanthi et al. 2011). In contrast to the apoptotic role of CHOP in AD and PD, constitutive overexpression of CHOP in myelinating cells under normal or ER stress conditions does not drive cell death (Southwood et al. 2016).

#### Cancer

CHOP expression in tumors correlates with stage, malignancy, and low survival in patients (Kim et al. 2012; Dalton et al. 2013). The incidence of K-ras(G12V)-induced lung cancer is markedly enhanced in the absence of CHOP (Huber et al. 2013), suggesting an anti-cancer activity of CHOP. As a well-known proapoptotic gene, CHOP is considered a drug target for cancer (Schonthal 2013). Elevated expression of CHOP is observed in tumors after chemotherapy or as a consequence of uncontrolled growth of malignant cells (Schonthal 2013; Flaherty et al. 2014). Upon drug treatment, CHOP induction enhances apoptosis through transactivation of DR5 in human carcinoma (Yamaguchi and Wang 2004), prostate cancer (Shiraishi et al. 2005), pancreatic cancer (Abdelrahim et al. 2006), and lung cancer (Lin et al. 2008). CHOP also inhibits the expression of transferrin, a key protein for cell survival in hepatoma cells, decreasing tumor cell viability (You et al. 2003).

On the other hand, CHOP promotes hepatic carcinogenesis by enhancing inflammation, fibrosis, and cell death in the liver (DeZwaan-McCabe et al. 2013). In addition, *Chop*^−/−^ mice display smaller tumor nodules with reduced numbers of macrophages and levels of IFNγ. Since hepatocellular carcinoma is induced by chronic inflammation, CHOP may promote tumorigenesis by modulating the tumor microenvironment and macrophage recruitment to the tumor (Scaiewicz et al. 2013). Furthermore, *Chop* deficiency promotes the anti-tumor activity of tumor-infiltrating myeloid-derived suppressor cells (MDSC) by decreasing IL-6 and phospho-STAT3, delaying tumor progression (Thevenot et al. 2014). Unfortunately, many in vivo studies of CHOP use whole-body knockout mice, so it is not possible to understand the mechanistic basis for a phenotype.

Of all of the known TFs that function downstream from ER stress, CHOP is the only one that, when deleted, protects cells from cell death upon protein misfolding in the ER. However, the exact mechanism by which CHOP induces cell death remains unclear. Although several death-related genes are reported as targets of CHOP, they were not characterized by ChIP-seq analysis (Han et al. 2013a). This finding might result from different contexts of heterodimeric TFs that function with CHOP at different states or stages of differentiation or cancer, respectively. Therefore, it is essential to identify binding partners of CHOP to uncover unknown issues.

### XBP1

#### Metabolism

β-Cell-specific *Xbp1* deletion causes β-cell loss and reduces insulin content due to impaired proinsulin processing and constitutive hyperactivation of IRE1α that was proposed to degrade a subset of mRNAs encoding proinsulin processing enzymes (Lee et al. 2011a) in a process called IRE1α-dependent RNA degradation (RIDD) (Kaser et al. 2008; Han et al. 2009; Lee et al. 2011a). IRE1α hyperactivation typically occurs in cells that are deleted in XBP1. Developmental β-cell-specific *Ire1*α deletion also causes β-cell failure (Xu et al. 2014). However, *Ire1*α deletion in mature mice does reduce β-cell mass or expression of β-cell-specific genes, but glucose-stimulated proinsulin mRNA translation is defective primarily due to reduced glucose-stimulated induction of genes involved in proinsulin cotranslational translocation into the ER and signal peptide processing (Hassler et al. 2015). In contrast, sustained expression of XBP1s causes β-cell dysfunction and apoptosis through reduced expression of PDX1 and MAFA (Allagnat et al. 2010). Therefore, fine-tuning of XBP1s expression is necessary to maintain β-cell function.

In hepatocytes, XBP1s is required for glucose and lipid homeostasis. Hepatic overexpression of XBP1s suppresses gluconeogenesis through its interaction with FoxO1 to promote its degradation through the 26S proteasome system (Zhou et al. 2011). On the other hand, upon prolonged fasting, XBP1s directly induces expression of PPARα, the master regulator of the starvation response, leading to fatty acid β-oxidation and ketogenesis in the liver (Shao et al. 2014). XBP1s was reported to directly induce transcription of lipogenic genes in the liver, including *Dgat2*, *Scd1*, and *Acc2* (Lee et al. 2008). *Mx1-Cre* mediated *Xbp1* deletion in the liver causes profound defects in de novo hepatic lipogenesis, reducing serum TG, cholesterol, and free fatty acids. In contrast, lipogenesis is not induced, but very low-density lipoprotein (VLDL) assembly and secretion are impaired upon *Ire1*α Ad-Cre-mediated deletion in hepatocytes, causing hepatosteatosis and hypolipidemia (Zhang et al. 2011; Wang et al. 2012c). The defect in VLDL secretion is partly due to reduced expression of protein disulfide isomerase (PDI), a transcriptional target of XBP1s. PDI is essential for the activity of microtriglyceride transfer protein (MTP) that promotes TG uptake into the smooth ER (Wang et al. 2015). *Xbp1* deletion by *Mx1-Cre* also causes deletion in Kupffer cells (Lee et al. 2008), whereas Ad-Cre-mediated or albumin-Cre-mediated *Ire1*α deletion is restricted to hepatocytes, and this may explain why lipogenic gene expression is not reduced in the latter cases (Zhang et al. 2011; Wang et al. 2012c). This emphasizes that the particular Cre used for tissue-specific deletion may significantly impact the phenotype. Therefore, it is desirable to analyze deletion promoted by two independent Cre drivers. Consequently, it remains unknown how ER stress affects lipid accumulation or secretion through the IRE1α/XBP1 pathway.

XBP1s is also involved in insulin signaling in the liver. P85α and p85β, regulatory subunits of phosphoinositide 3 kinase (PI3K), interact with XBP1s and increase its nuclear translocation. *p85*α deletion in the liver reduces the UPR due to decreased XBP1s accumulation in the nucleus (Winnay et al. 2010). The interaction between p85α and XBP1s is lost in leptin-deficient *ob/ob* mice, causing defective nuclear XBP1s translocation and an inability to resolve ER stress in obese mice (Park et al. 2010b).

#### Inflammation

XBP1 was first discovered as a regulatory protein that binds to the X-box element within the human major histocompatibility complex (MHC) class II locus (Liou et al. 1988). Deletion of *Ire1*α or *Xbp1* in the lymphoid system impairs adaptive immune responses, especially plasma cell differentiation responsible for antibody production (Reimold et al. 2001; Tirosh et al. 2005; Zhang et al. 2005), and decreases dendritic cells (Iwakoshi et al. 2007). In addition, XBP1 induces expression of the major lineage determinant GATA-1 that is necessary for eosinophil, but not basophil or neutrophil, differentiation (Bettigole et al. 2015), indicating its pivotal role in immune cell development.

XBP1s is also actively involved in inflammation. *Xbp1* deletion in intestinal epithelial cells (IECs) causes ER stress and defects in Paneth cell function, leading to spontaneous enteritis and increased susceptibility to colitis (Kaser et al. 2008; Adolph et al. 2013). However, IEC-specific *Ire1*α deletion does not cause defects in Paneth cells or intestinal dysplasia and even corrects defects in *Xbp1*-null Paneth cells. Therefore, *Xbp1* deletion causes hyperactivation of IRE1α, leading to intestinal defects. In macrophages, TLR4 and TLR2 activate the IRE1α–XBP1 pathway, leading to sustained production of proinflammatory cytokines (Martinon et al. 2010). Indeed, myeloid-specific *Ire1*α deletion reduces inflammatory responses in a murine model of rheumatoid arthritis; however, IRE1α activation did not occur as a response to ER stress but rather TLR activation (Qiu et al. 2013).

#### Neurodegenerative disease

Developmental *Xbp1* deletion in the murine nervous system protects dopaminergic neurons from 6-OHDA treatment (Valdes et al. 2014), suggesting a pathogenic role for XBP1 in PD pathology. Therefore, a low-level UPR activation may produce an adaptive response during neuronal development to maintain protein homeostasis in the absence of XBP1 signaling. In contrast, down-regulation of XBP1 expression in adult substantia nigra pars compacta (SNpc) induces strong ER stress that triggers massive dopaminergic neuron degeneration. In addition, delivery of XBP1s into the SNpc of adult mice protects dopaminergic neurons from 6-OHDA (Valdes et al. 2014). Similar to PD, XBP1 suppresses Aβ neurotoxicity in the *Drosophila* eye and in cultured neurons by attenuating expression of the ryanodine receptor RyR3 to decrease Ca^2+^ release into the cytosol (Casas-Tinto et al. 2011). *Xbp1* deletion delays progression of Huntington's disease (HD) by increasing autophagy to degrade the mutant Huntingtin (Htt) protein. XBP deficiency promotes autophagy by augmenting expression of FoxO1, encoding a key TF for autophagy in neurons (Vidal et al. 2012). However, viral delivery of XBP1s into the striatum reduced mutant Htt protein aggregation in HD mice (Zuleta et al. 2012). These findings again suggest that an optimal level of XBP1s expression is essential to promote mutant Htt proper folding and prevent aggregation. In the SOD1^G93A^ transgenic ALS mouse model, ER stress and XBP1s induction are observed. XBP1 deficiency in motor neurons of SOD1^G93A^ mice also increases autophagy to clear mutant SOD1 aggregates, suggesting that XBP1s may contribute to ALS pathogenesis (Hetz et al. 2009). Therefore, XBP1s has protective or detrimental effects on neurodegeneration, although the exact mechanism remains to be elucidated (Hetz and Saxena 2017).

#### Cancer

Elevated XBP1s expression is observed in many human tumors, including breast cancer (Davies et al. 2008; Chen et al. 2014), pancreatic adenocarcinomas (Romero-Ramirez et al. 2009), multiple myeloma (Carrasco et al. 2007), chronic lymphocytic leukemia (CLL) (Krysov et al. 2014), and plasma cell malignancy (Maestre et al. 2009), suggesting that *Xbp1* is a proto-oncogene. Consistently, myeloma patients with higher amounts of XBP1s have a poorer overall survival (Bagratuni et al. 2010), and the growth of *Xbp1*-deficient tumor cells is impaired in xenograft models (Romero-Ramirez et al. 2004). XBP1s also promotes tumorigenesis by assembling a transcription complex with HIF1α to transactivate target genes (Chen et al. 2014) and up-regulating Snail expression to induce epithelial-to-mesenchymal transition (EMT) (Li et al. 2015). XBP1s in tumor-associated dendritic cells promotes ovarian cancer by inhibiting anti-tumor immunity through abnormal lipid accumulation in tumor-associated dendritic cells (Cubillos-Ruiz et al. 2015). In contrast, *Xbp1*-deficient IECs exhibit increased turnover through NFκB-dependent activation of STAT3, promoting colitis-associated cancer and spontaneous adenomatous polyposis coli (APC)-related tumors in mice (Niederreiter et al. 2013), suggesting that XBP1 might act as a tumor suppressor in the intestine.

As the most ancient UPR TF, XBP1 has a critical role in both physiological and pathological states. The activity of XBP1s is affected by either ER stress or other stimuli such as the insulin signaling pathway (Park et al. 2010b; Winnay et al. 2010) or activation of pattern recognition receptors (Martinon et al. 2010; Qiu et al. 2013). This observation implies that this ancient TF might act as a nexus for environmental stimuli besides ER stress. This new concept remains to be elucidated.

### Additional TFs

Several additional TFs are regulated by ER stress and the UPR through transcriptional, translational, or post-translational controls.

#### ATF5

ATF4 activates transcription of ATF5 (Zhou et al. 2008), which was first cloned as a factor in developing sensory neurons of the olfactory epithelium (Hansen et al. 2002). ATF5 inhibits differentiation of neuroprogenitor cells into neurons (Angelastro et al. 2003) and astrocytes (Angelastro et al. 2005) and of oligodendrocyte precursors into oligodendrocytes (Mason et al. 2005). In contrast, *Atf5*^−/−^ mice exhibit massive reduction in mature olfactory sensory neurons (OSNs), and ectopic expression of ATF5 in neural progenitor cells induces expression of multiple OSN-specific genes, suggesting that ATF5 promotes OSN differentiation (Wang et al. 2012d). ATF5 also promotes survival of malignant cells by stimulating expression of anti-apoptotic B-cell leukemia-2 (BCL2) and myeloid cell leukemia sequence-1 (MCL1), a BCL2 family member (Sheng et al. 2010; Dluzen et al. 2011), indicating a prosurvival role in cancer. In addition, polymorphisms located in the promoter region impact ATF5 expression. Increased ATF5 expression induces asparagine synthetase in acute lymphoblastic leukemia and reduces therapeutic treatment with L-asparaginase (Rousseau et al. 2011). ATF5 can promote inflammatory responses upon ER stress in β cells by increasing transcription of thioredoxin-interacting protein (TXNIP) to activate the NLRP3 inflammasome to produce IL-1β (Oslowski et al. 2012).

#### NF-κB

Many studies support the notion that ER stress stimulates inflammatory responses though activation of NK-κB. Various ER stress-inducing agents increase the DNA-binding activity of NF-κB as well as downstream target gene expression (Pahl and Baeuerle 1995). ER stress is proposed to activate NF-κB through several mechanisms. ER stress-induced NF-κB activation is impaired in *Ire1a* knockdown cells and *Ire1*α^−/−^ cells due to loss of an IRE1α and IκB kinase complex (Hu et al. 2006). In addition, genetic and pharmacological inhibition of ATF6α attenuates NF-κB activation, suggesting a stimulatory role for ATF6α in NF-κB signaling (Yamazaki et al. 2009). eIF2α phosphorylation also activates NF-κB by inhibiting the synthesis of the short-lived inhibitor of NF-κB, IκBα (Deng et al. 2002; Jiang et al. 2003). It is essential to provide definitive evidence for a biochemical link between ER stress sensor (IRE1α, PERK, and ATF6α) activation and subsequent downstream inflammatory responses.

In contrast, chronic ER stress inhibits NF-κB activity (Hayakawa et al. 2009). Furthermore, preconditioning with ER stress markedly inhibits expression of NF-κB target cytokines through up-regulation of C/EBPβ (Du et al. 2009). This phenomenon is mediated by up-regulation of the ubiquitin-editing enzyme A20 (also known as *TNFAIP3*) upon ER stress, which is an endogenous negative regulator of NF-κB (Nakajima et al. 2010).

#### CREBH

CREBH, encoded by *CREB3L3*, is a hepatocyte-specific TF that was originally identified as a central regulator of the acute phase response (Zhang et al. 2006). As a mediator of inflammatory responses in the liver, CREBH controls hepatic lipid metabolism under metabolic stress conditions (Zhang et al. 2012). Inflammatory cytokines induce transcription of CREBH, and ER stress stimulates its cleavage and activation by S1P and S2P. CREBH promotes expression of genes encoding functions in de novo lipogenesis, TG and cholesterol biosynthesis, fatty acid elongation and oxidation, lipolysis, and lipid transport. In addition, CREBH activates expression of Fsp27, a lipid droplet-associated protein (Xu et al. 2015). Consistently, forced expression of CREBH in the liver causes hepatic lipid accumulation, although TG levels in the blood decrease (Zhang et al. 2012; Xu et al. 2015). In addition, CREBH promotes expression of lipoprotein lipase (Lpl) coactivators apolipoprotein C2 (*Apoc2*), *Apoa4*, and *Apoa5* and concurrently down-regulates Lpl inhibitor Apoc3 (Lee et al. 2011b). As a result, *Crebl3*^−/−^ mice display hypertriglyceridemia due to inefficient TG clearance. Furthermore, multiple nonsynonymous mutations in *CREB3L3* are associated with extreme hypertriglyceridemia, suggesting a pivotal role of CREBH in human TG metabolism (Lee et al. 2011b). In addition to lipid metabolism, CREBH promotes hepatic gluconeogenesis by inducing expression of gluconeogenic enzyme in a CRTC2-dependent manner (Lee et al. 2010b). Consistently, knockdown of CREBH improves fasting hyperglycemia in diabetic *db/db* mice, suggesting that CREBH is a critical regulator for hepatic gluconeogenesis.

## Integrated response of UPR TFs

Here we described the function of each UPR-associated TF in multiple cellular pathways and associated diseases. However, it is also important to consider that bZIP TFs bind DNA as either homodimers or heterodimers. Their partners could be other UPR TFs or different TFs with no function in the UPR. For example, expression of ATF4 target genes is enhanced when it heterodimerizes with CHOP upon ER stress (Han et al. 2013a). XBP1u translated from mammalian unspliced XBP1 mRNA acts as negative regulator by heterodimerizing with XBP1s to promote its degradation (Yoshida et al. 2006). In addition, ATF6α/XBP1 and CREBH/ATF6α heterodimers possess greater transcriptional activity than either respective homodimer (Zhang et al. 2006; Yamamoto et al. 2007).

The UPR TFs also form heterodimers with the other TFs with no or little function in the UPR. ATF4 forms heterodimers with nuclear factor-like 2 (NRF2) and C/EBPγ upon oxidative stress to activate transcription of antioxidant genes (He et al. 2001; Huggins et al. 2015). Heterodimers of CHOP and C/EBPβ inhibit adipogenesis (Tang and Lane 2000). In addition, insulin signaling disrupts p85α–p85β heterodimers so that p85 can interact with XBP1s to facilitate its nuclear translocation and induce UPR transcription (Park et al. 2010b). These results strongly suggest that combinatorial interactions of TFs may generate diverse responses to different stimuli in different cell types.

## Therapeutic implications

Given the role of UPR-induced TFs across a range of human diseases, there is great interest in pharmacologically modulating their activity to control ER stress-mediated diseases. There are two approaches to modulate TF activity. The first is to develop small molecules that can directly bind and modulate TF function. The second is to modulate effectors upstream of or downstream from the TFs.

TFs are generally considered to be poor drug targets due to the inability of small molecules to block protein–protein and protein–DNA binding interfaces (Imming et al. 2006). Although chemical genomics provides examples of small molecules that can modulate the activity of TFs, until now, few small molecules were reported to directly bind and inhibit TFs. Nevertheless, several studies identified small molecules that modulate the activity or expression UPR TFs. For example, E235 was identified through the screening of small molecules that activate ATF4 expression in human fibrosarcoma HT1080 cells (Sayers et al. 2013). E235 treatment increases the levels of phosphorylated eIF2α without induction of XBP1 splicing. E235 decreases viability in several mouse and human cell lines, which is abolished by knockdown of ATF4, suggesting that this drug acts specifically on eIF2α/ATF4. Another small molecule, ML291, was developed through a high-throughput screen of the National Institutes of Health Molecular Libraries Small Molecule Repository (MLSMR) (Flaherty et al. 2010). This molecule selectively activates the eIF2α/ATF4 pathway but not the IRE1α or ATF6α pathway. This molecule induces cell death in a CHOP-dependent manner in a number of cell lines, and there is enthusiastic support to develop this molecule for cancer therapy. A recent study also suggests that modulation of ER stress could be a selective target for cancer cells that undergo EMT (Feng et al. 2014). During EMT, cells secrete more secretory molecules, such as extracellular matrix proteins, which provokes eIF2α phosphorylation and subsequent ATF4 induction. Thus, cells undergoing EMT are more sensitive to ER stress compared with cells without EMT. This selective toxicity of cells stressed by a harsh environment or protein misfolding offers a selective advantage to using these agents to uniquely destroy tumor cells (Feng et al. 2014).

For XBP1, several small molecules have been developed recently (Obacz et al. 2017). STF-083010 (Papandreou et al. 2011), salicylaldehydes (Volkmann et al. 2011), 4µ8C (Cross et al. 2012), compound 3 (Wang et al. 2012b), and quercetin (Wiseman et al. 2010) exert their effect on XBP1 by modulating IRE1α activity. Although XBP1 mRNA is the only splicing substrate for IRE1α, targeting IRE1α activity might cause unknown adverse effects due to RIDD. In contrast to these molecules, toyocamycin, a nucleoside-type antibiotic analog of adenosine, blocks chemically induced XBP1 splicing as well as XBP1 target gene expression without affecting IRE1α phosphorylation (Ri et al. 2012). MKC-3946, a salicylaldehydes derivative, inhibits chemically induced XBP1 splicing in multiple myeloma cell lines as well as patient-derived samples without affecting IRE1α phosphorylation in this context (Mimura et al. 2012). In addition, other small molecules, including MKC9989, OICR464, and OICR573, block XBP1 splicing with minimal effect on IRE1α kinase activity, suggesting a direct effect on XBP1 (Sanches et al. 2014).

Another approach to modulate UPR TFs is to use small molecules that can inhibit upstream factors. For example, GSK2656157, an ATP-competitive inhibitor of PERK, suppresses eIF2α phosphorylation and decreases ATF4 and CHOP expression through inhibition of stress-induced PERK autophosphorylation (Atkins et al. 2013). However, the effects of GSK2656157 are not solely dependent on PERK and eIF2α phosphorylation (Krishnamoorthy et al. 2014). Another PERK inhibitor, GSK2606414, gave new insight into how this small molecule can be used in human disease. Prion disease, which is caused by accumulation of misfolded prion protein (PrP) due to prion replication, causes sustained activation of the PERK/eIF2α pathway (Moreno et al. 2012). Oral treatment with GSK2606414 prevented UPR-mediated translational attenuation and abrogated development of prion diseases in mice (Moreno et al. 2013). Importantly, this molecule can penetrate the blood–brain barrier, showing therapeutic potential for brain disease.

Although there have been advances in the development of small molecules to target UPR TFs for therapeutic application, there must be some cautionary considerations for this approach. First, the expression levels of TFs need to be properly regulated at the appropriate level. For example, the absence of ATF6α causes liver steatosis upon ER stress (Wu et al. 2007; Rutkowski et al. 2008; Yamamoto et al. 2010), whereas overexpression of the active form of ATF6α in zebrafish livers causes fatty liver due to lipid accumulation (Howarth et al. 2014). There appears to be an optimum of expression versus toxicity. This is an essential feature that needs further investigation. Spatial differences in expression are another aspect that needs to be considered. Since the expression of some proteins is essential for some cells, such as PERK in pancreatic β cells, the impact of inhibiting their expression in other tissues needs further investigation. Additionally, expression of ATF4 in the hypothalamus induces insulin resistance, whereas ATF4 expression in muscle protects against diet-induced insulin resistance, suggesting that even the same TF exerts responses depending on when and where it is expressed. This emphasizes the requirement to target selective UPR agonist/antagonist pathways in selective cell types. If these two technologies are blended together, it will be beneficial to modulate UPR signaling to ameliorate disease progression.

Alternatively, it can be envisioned that targeting UPR signaling may be very selective to those cells that experience ER stress; i.e., virally infected cells, cells exposed to toxic compounds, and transformed cells. Thus, there is tremendous potential to selectively target “stressed” cells versus normal cells.

## Perspectives

The UPR is a set of highly conserved signal transduction pathways activated when ER homeostasis is disturbed, referred to as ER stress. The ultimate step in the UPR involves activation of a set of bZIP-containing TFs that coordinate adaptive or cell death responses. Numerous signal transduction events and TFs are known that signal the UPR, although their significance and roles in physiology and pathophysiology remain largely unknown. Although the primary role of these TFs is to restore ER homeostasis, new lines of evidence suggest that they provide functions in other physiological or pathological processes, including immune responses, cancer development, and insulin signaling. The diverse function of each TF activated by the UPR might be due to their characteristic to form heterodimers with different partners at different times or in response to different stimuli. Thus, it is essential to identify the process by which these TFs network to affect or interact with other TFs. Nevertheless, targeting the TFs is an attractive approach to treat ER stress-mediated human disease. However, it is not known how these TFs function in the absence of ER stress, but, based on the significant phenotypes observed upon their deletion, it is likely that they function in cell-type-specific networks in many aspects of cell physiology. Identifying the role of TFs associated with ER stress and their role in the absence of ER stress will provide novel insights for future investigations to characterize the mechanism and functionality toward development of therapeutic applications for many disease states.
